# Supplementary material for: Pre-touch reaction is preferred over post-touch reaction in interaction with displayed agent
Source: PeerJ Comput Sci. 2024 Aug 23;10:e2277. doi: 10.7717/peerj-cs.2277 (PMC11419639; doi:10.7717/peerj-cs.2277)
Supplement: Supplemental Information 2 [file peerj-cs-10-2277-s002.docx]

Questionnaire (translated from Japanese)

Please let us know your impressions of this agent.

Please select a number from 1 to 7 according to the level of the axis written on it.

| 1 | This agent is… | Fake ＿＿＿＿＿＿ 　　Natural |
| --- | --- | --- |
| 2 | This agent is… | Machinelike ＿＿＿＿＿＿　Humanlike |
| 3 | This agent is… | Unconscious ＿＿＿＿＿＿ Conscious |
| 4 | This agent is… | Artificial ＿＿＿＿＿＿ 　　Lifelike |
| 5 | This agent is moving | rigidly ＿＿＿＿＿＿ elegantly |
|  |  |  |
| 6 | This agent is… | Dislike ＿＿＿＿＿＿ 　　　Like |
| 7 | This agent is… | Unfriendly ＿＿＿＿＿＿ 　Friendly |
| 8 | This agent is… | Unkind ＿＿＿＿＿＿ 　　Kind |
| 9 | This agent is… | Unpleasant ＿＿＿＿＿＿ Pleasant |
| 10 | This agent is… | Awful＿＿＿＿＿＿ 　　　Nice |
|  |  |  |
| 11 | This agent’s reaction is human-like | disagree ＿＿＿＿＿＿ agree |
| 12 | The reaction distance is natural | disagree ＿＿＿＿＿＿ agree |
| 13 | In total, this agent is good | disagree ＿＿＿＿＿＿ agree |
|  |  |  |

Please enter any other comments you may have.
